# Obesity trajectories and risk of dementia: 28 years of follow-up in the Whitehall II Study

**DOI:** 10.1016/j.jalz.2017.06.2637

**Published:** 2018-02

**Authors:** Archana Singh-Manoux, Aline Dugravot, Martin Shipley, Eric J. Brunner, Alexis Elbaz, Séverine Sabia, Mika Kivimaki

**Affiliations:** a INSERM, U1018, Centre for Research in Epidemiology and Population Health, Villejuif, France; b Department of Epidemiology and Public Health, University College London, UK

**Keywords:** Obesity, BMI, Dementia, Waist circumference, Waist-to-hip ratio

## Abstract

**Introduction:**

We examined whether obesity at ages 50, 60, and 70 years is associated with subsequent dementia. Changes in body mass index (BMI) for more than 28 years before dementia diagnosis were compared with changes in BMI in those free of dementia.

**Methods:**

A total of 10,308 adults (33% women) aged 35 to 55 years in 1985 were followed up until 2015. BMI was assessed six times and 329 cases of dementia were recorded.

**Results:**

Obesity (BMI ≥30 kg/m^2^) at age 50 years (hazard ratio = 1.93; 1.35–2.75) but not at 60 or 70 years was associated with risk of dementia. Trajectories of BMI differed in those with dementia compared with all others (*P* < .0001) or to matched control subjects (*P* < .0001) such that BMI in dementia cases was higher from 28 years (*P* = .001) to 16 years (*P* = .05) and lower starting 8 years before diagnosis.

**Discussion:**

Obesity in midlife and weight loss in the preclinical phase characterizes dementia; the current obesity epidemic may affect future dementia rates.

## 1 Introduction

Prospective studies, particularly those with extended follow-up [1], suggest midlife obesity is a risk factor for dementia [2], [3], [4], [5]. Meta-analyses and systematic reviews show the excess risk to be similar for Alzheimer's disease and all-cause dementia [6], [7], [8], with effects being stronger in studies with a follow-up longer than 10 years, and when body mass index (BMI) is assessed before age 60 years [6].

The hypothesis that midlife obesity increases risk for dementia has been challenged by two sets of recent findings. The first using electronic patient records from 2 million adults showed lower rates of dementia in the obese and progressively decreasing risk with increasing obesity [9]. As previous meta-analyses suggesting midlife obesity to be a risk factor for dementia are based on less than 50,000 persons overall [2], [6], [7], inclusion of this new study in future meta-analyses would undoubtedly change conclusions. The second set of studies was based on a Mendelian randomization (MR) approach, which uses genetic variants as proxies for exposures, to show no association between obesity and dementia [10], [11]. These findings taken together raise questions about the inclusion of midlife obesity in guidelines for dementia prevention, such as the one published last year [12].

BMI in older adults with dementia is typically lower than age peers, with weight loss starting 10 years or more before clinical onset [13]. Pathophysiological processes underlying dementia are known to span several years, perhaps decades [14], [15], and may lead to weight loss before disease onset [16]. However, the BMI trajectory in those with dementia remains poorly characterized. The few studies with repeat BMI data over the adult life course show those with dementia to gain less weight [17], or experience accelerated weight loss in old age [13], [18], but no differences in BMI in midlife. The inconsistencies are likely to be because of the analysis of change in BMI being anchored by wave of BMI assessment rather than the etiologic stage of dementia.

We use an innovative analytic approach with two objectives. First, to understand when in midlife obesity carries risk for dementia, we model the risk associated with obesity at ages 50, 60, and 70 years. This is in contrast to studies that used a wide age range and adjusted for age to make inferences about the effect of “midlife” obesity. Our method allows better insight into the manner in which age modifies the association between obesity and dementia. Second, to study changes in BMI, we use repeat data and model trajectories of BMI for more than 28 years, using a backward timescale anchored to the year of dementia diagnosis. This method allows BMI differences between dementia cases and those free of dementia to be estimated for each of the 28 years preceding dementia diagnosis. Analyses were repeated with waist circumference and waist-to-hip ratio to determine whether their associations with dementia are similar to that obtained for BMI.

## 2 Methods

### 2.1 Study design and participants

The Whitehall II study is an ongoing cohort study of men and women originally employed by the British civil service in London-based offices [19]. A total of 10,308 persons (6895 men and 3413 women, aged 35–55) were recruited in the study from the years 1985 to 1988, with a response rate of 73%. Since the baseline medical examination, follow-up examinations have taken place approximately every 5 years. Data collection involves a postal questionnaire (health behaviors, psychosocial factors, and mental health) and a medical examination undertaken by trained nurses using a standard protocol at a central London clinic or at home for those unable/unwilling to travel. Written informed consent from participants and research ethics approvals were renewed at each contact; the most recent approval was from the University College London Hospital Committee on the Ethics of Human Research, reference number 85/0938.

### 2.2 Measurement of adiposity

We obtained data for BMI (weight in kilograms/height in meters squared) from study baseline 1985 to 1988 and all subsequent clinical assessments; flow chart of study is presented in Supplementary Fig. 1. Weight was measured in underwear to the nearest 0.1 kg on Soehnle electronic scales with digital readout (Leifheit AS, Nassau, Germany). Height was measured in bare feet to the nearest 1 mm using a stadiometer with the participant standing erect with head in the Frankfurt plane. The World Health Organization classification was used to categorize BMI as <18.5 kg/m^2^ (underweight), 18.5 to 24.99 kg/m^2^ (normal weight), 25 to 29.99 kg/m^2^ (overweight), and ≥30 kg/m^2^ (obese) [20].

Waist and hip circumferences, starting at the 1991 to 1993 assessment, were measured with subjects in the standing position in light clothing, using a fiberglass tape measure at 600 g tension. Waist circumference was taken as the smallest circumference at or below the costal margin and hip circumference at the level of the greater trochanter. Waist circumference categories were small (≤94/80 cm in men/women), intermediate (94 to ≤102/80 to ≤88 cm in men/women), and large (≥102/88 cm in men/women) [21]. For waist-to-hip ratio, we used values ≥1.0 in men and 0.85 in women to denote obesity [22].

### 2.3 Dementia

We used comprehensive tracing of electronic health records for dementia ascertainment using three databases: the national hospital episode statistics database, the Mental Health Services Data Set, and the national mortality register. The National Health Service in the UK uses in-house codes mapped onto International Classification of Diseases-10 codes for dementia. The National Health Service provides most of the health care; hospital episode statistics and Mental Health Services Data Set are national databases with information on both inpatient and outpatient care, with the latter also including data on care in the community. Record linkage until March 31, 2015 identified 329 cases of dementia, 176 cases were first recorded in the hospitalization register, 145 in the mental health register, and eight in the mortality register.

The validity of dementia cases in our study is supported by modeling changes in the global cognitive score, composed of tests of memory, reasoning, and phonemic and semantic fluency administered to the participants in 1997, 2003, 2007, and 2012 [23]. These results show accelerated decline in global cognitive score in the 8 to 10 years before dementia diagnosis (Supplementary Fig. 2) as has been shown in studies that use a “gold-standard” dementia ascertainment procedure [24].

### 2.4 Covariates

*Education* was reported by the participant as the highest level of education achieved and regrouped into five standard hierarchic levels: no formal education, lower secondary education, higher secondary education, university degree, and higher university degree.

*Cardiovascular disease (CVD), diabetes, and medication*: ascertainment of these conditions was based on two methods: study specific assessments (1985, 1989, 1991, 1997, 2001, 2003, 2006, 2007, and 2012) and linkage to electronic health records for nonresponders and chronic conditions occurring between study waves. A 12-lead resting electrocardiogram recording, coded using the Minnesota system, was used for coronary heart disease. Stroke assessment was based on the Multinational MONItoring of trends and determinants in CArdiovascular disease (MONICA)-Ausburg stroke questionnaire, corroborated in medical records. Diabetes was determined by fasting glucose ≥7.0 mmol/L, reported doctor-diagnosed diabetes, or use of diabetes medication. Medication for CVD was reported by the participants at each wave.

### 2.5 Statistical analysis

We undertook two sets of analyses; first, Cox regression to examine associations of adiposity measures at ages 50, 60, and 70 years (three separate models) with subsequent dementia. Analyses were based on all participants with data on obesity, follow-up for dementia commencing on the date of the obesity measure. Second, we compared changes in BMI (trajectories) in those with dementia with other participants using a backward timescale. The trajectory analysis was repeated using a case (dementia cases, *n* = 329) and control (*n* = 1974) design to better control for the confounding effects of age, sex, education, and period effects in measures of BMI and dementia.

#### 2.5.1 Analysis 1: associations of adiposity at ages 50, 60, and 70 years with dementia and mortality

Age ranges of the participants at the six clinical evaluations between 1985 to 1988 and 2012 to 2013 were 35 to 55, 40 to 64, 45 to 69, 50 to 74, 55 to 79, and 60 to 84 years. We extracted data on adiposity measures at ages 50, 60, and 70 years for each participant across the data waves. For example, a participant who was 50 years old at the first clinical assessment (1985–1988) contributed to the analysis (and person years of follow-up) for “BMI at age 50” using data from the first wave, the analyses for “BMI at age 60” using data from the third clinic (1997–1999), and “BMI at age 70” using data from the fifth clinic (2007–2009). The analyses were first run with dementia as an outcome and repeated with mortality; both using Cox regression with date of entry being the date of clinical assessment from which the obesity measure (separate models for age 50, 60, and 70 years) was drawn. In the analysis for dementia, those who died free of dementia were censored at death so that participants were followed until the record of dementia, death, or March 31, 2015, whichever came first. For analysis of mortality, they were followed until the record of death or March 31, 2015, whichever came first. All analyses were adjusted for age, sex, and education. In Supplementary Tables, we show analyses with further adjustment for diabetes, CVD, and medication for these conditions.

#### 2.5.2 Analysis 2: trajectories of adiposity before dementia

Trajectories of BMI for more than 28 years were modeled using a backward timescale such that year 0 was year of dementia for dementia cases, year of death for those who died during the follow-up, and March 31, 2015 (end of follow-up) for all others. BMI in each of the preceding 28 years (years 0 to −28) was estimated from mixed effects models with the intercept and slope as random effects and a backward timescale [24]. Dementia (coded as 1 for cases and 0 for others) and its interaction with time and time squared (to allow for nonlinear change) were added to the model to test for differences in BMI trajectories between cases and others. This modeling strategy implies that year 0 (the index date) was the intercept in the analysis and the beta associated with the dementia term yielded the difference in BMI between cases and others/control subjects at dementia diagnosis. The slope terms (time and time squared) allow the assessment of changes in BMI for more than the previous 28 years. To test whether these were different in cases and others, we examined whether the terms dementia × time and dementia × time squared improved the fit of the model using the Wald test. Analyses were adjusted for age, sex, education, their interactions with time and time squared, and for 5-year birth cohort to take cohort effects into account.

These analyses on BMI trajectories anchored to the year of dementia diagnosis were repeated using a case-control approach to account for period effects in measures of BMI and dementia. Year 0 for both cases and control subjects was the year of dementia diagnosis. Each case was individually matched to six control subjects drawn randomly from the study population using the following criteria: age (5-year age group at the index year), sex, education (university degree or not), being alive at year 0, and without a diagnosis of dementia at the end of follow-up. The case-control analyses were also undertaken for waist circumference and waist-to-hip ratio on the same cases and control subjects, albeit with a shorter follow-up of 22 years.

The software SAS 9.4 (SAS Institute, NC, USA) was used for data management and STATA 14 (StataCorp LP, College Station, TX, USA) for analysis. A two-sided *P* value <.05 was considered statistically significant.

## 3 Results

The mean (standard deviation) age of those with dementia (N = 329) was 75.0 (5.4) years and those who died (N = 1653) was 67.6 (9.4) years. Cases of dementia accrued between 1995 and 2015, with 73% of cases recorded in the last 5 years of follow-up. Increasing age (hazard ratio [HR] for 1 year greater age at study baseline associated with a 1.21; 95% confidence interval, 1.19–1.24), female sex (HR = 1.58; 1.27–1.96), and education less than secondary school diploma (HR = 1.76; 1.41–2.19) were associated with higher risk of dementia. Table 1 presents study characteristics as a function of dementia status in the total population and in cases and control subjects. BMI in those with dementia was higher at age 50 years (*P* ≤ .01) but not different from those without dementia at age 60 and 70 years.

**Table 1:** Sample characteristics by dementia status at the end of follow-up

Characteristics	Total population			Case-control design		
	Dementia	No dementia	*P* value	Dementia	No dementia	*P* value
N	329	9974		329	1974	
Age at baseline, M (SD)	50.5 (4.5)	44.8 (6.0)	<.0001	50.5 (4.5)	49.2 (4.9)	<.0001
Sex, N (%) women	144 (43.8)	3267 (32.8)	<.0001	144 (43.8)	864 (43.8)	1
Education, N (%)						
No	52 (15.8)	977 (9.8)	<.0001	52 (15.8)	337 (17.1)	.21
Primary	146 (44.4)	3722 (37.3)		146 (44.4)	767 (38.9)	
A level	68 (20.7)	2675 (26.8)		68 (20.7)	492 (24.9)	
University or higher	63 (19.2)	2600 (26.1)		63 (19.2)	378 (19.2)	
Mean follow-up, M (SD)	24.5 (3.5)	26.7 (4.5)	<.0001	24.5 (3.5)	28.6 (1.1)	<.0001
BMI (kg/m^2^), M (SD)						
BMI at 50 years	26.1 (4.2)	25.5 (3.8)	.01	26.1 (4.2)	25.2 (3.6)	.0003
BMI at 60 years	26.4 (4.4)	26.5 (4.3)	.79	26.4 (4.4)	26.1 (4.1)	.32
BMI at 70 years	26.3 (4.6)	26.8 (4.4)	.18	26.3 (4.6)	26.7 (4.4)	.30

Abbreviations: BMI, body mass index; SD, standard deviation.

### 3.1 Associations of adiposity at ages 50, 60, and 70 years with dementia and mortality

A total of 10.9% of participants were obese at age 50, 17.1% at age 60, and 18.7% at age 70 years (Table 2). BMI ≥30 kg/m^2^ was associated with higher risk of dementia at age 50 (HR = 1.93; 1.35–2.75; *P* < .0001) but not at age 60 (HR = 1.31; 0.89–1.92; *P* = .17) or 70 years (HR = 0.87; 0.56–1.34; *P* = .53). BMI ≥30 kg/m^2^, at all three ages, was associated with greater risk of mortality. Our analyses were underpowered to examine the impact of underweight (BMI <18.5 kg/m^2^) on dementia but suggested increased risk of mortality in all age groups. Further adjustment for CVD, diabetes, and CVD medication did not alter these results (Supplementary Table 1). Analyses of waist circumference and waist-to-hip ratio are shown in Supplementary Tables 2 and 3 in the minimally and fully adjusted models. There was some evidence of an association between a large waist circumference at age 50 years and dementia (HR = 1.80; 0.98–3.33; *P* = .06) but not at ages 60 and 70 years (Supplementary Table 2); waist-to-hip ratio was not associated with dementia.

**Table 2:** Association of BMI at ages 50, 60, and 70 years with subsequent dementia and mortality∗

BMI	Dementia			Mortality		
	N cases/N	HR (95% CI)	*P* value	N cases/N	HR (95% CI)	*P* value
BMI at 50 years						
<18.5 kg/m^2^	1/72	0.51 (0.07–3.63)	.50	13/72	1.40 (0.81–2.43)	.23
18.5–24.9 kg/m^2^	128/4208	1.00	Ref	611/4208	1.00	Ref
25–29.9 kg/m^2^	104/3219	1.18 (0.91–1.52)	.22	505/3219	1.12 (0.99–1.26)	.06
≥30 kg/m^2^	42/913	1.93 (1.35–2.75)	<.0001	176/913	1.63 (1.38–1.94)	<.0001
BMI at 60 years						
<18.5 kg/m^2^	0/63	NA		12/63	2.51 (1.41–4.48)	.002
18.5–24.9 kg/m^2^	98/2819	1	Ref	343/2819	1.00	Ref
25–29.9 kg/m^2^	96/3162	1.02 (0.77–1.36)	.87	388/3162	1.13 (0.97–1.30)	.11
≥30 kg/m^2^	38/1246	1.31 (0.89–1.92)	.17	169/1246	1.53 (1.27–1.85)	<.0001
BMI at 70 years						
<18.5 kg/m^2^	2/39	1.89 (0.46–7.81)	.37	6/39	2.54 (1.12–5.79)	.03
18.5–24.9 kg/m^2^	72/1731	1.00	Ref	166/1734	1.00	Ref
25–29.9 kg/m^2^	57/2159	0.61 (0.43–0.87)	.006	196/2160	0.93 (0.76–1.15)	.52
≥30 kg/m^2^	30/904	0.87 (0.56–1.34)	.53	102/907	1.42 (1.10–1.82)	.007

Abbreviations: BMI, body mass index; CI, confidence interval; HR, hazard ratio; SD, standard deviation.

∗ Analyses adjusted for age, sex, and education.

### 3.2 Trajectories of adiposity before dementia

**Fig. 1:**
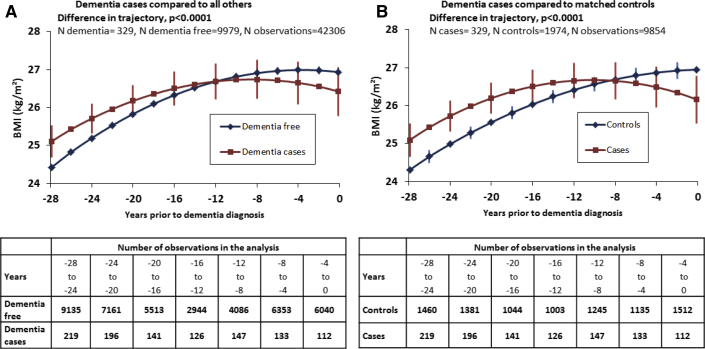
Trajectories of BMI in the 28 years before dementia. (1) The figure represents marginal effects of dementia on trajectories of BMI, adjusted for age, sex, and education and 5-year birth cohort in panel (A). Mean number of observations per participant is 3.3 for dementia cases and 4.1 for those dementia free for approach (A) and 4.4 for control subjects for approach (B). (2) The 95% confidence intervals are presented for alternate time points for ease of presentation.

The trajectory of BMI for more than 28 years was different (*P* < .0001) in those with dementia compared with all nondemented participants (Fig. 1A), the differences in BMI between these two groups are presented in Table 3. These results, using a backward timescale, showed that those with dementia had higher BMI in midlife and accelerated decline in the decade before dementia diagnosis. Analyses with the backward timescale were repeated using a case-control design (Fig. 1B). These analyses also show BMI trajectories to be different in cases and control subjects (*P* < .0001). In cases BMI was higher in midlife and they experienced accelerated decline in BMI in the years before dementia. BMI in cases was significantly higher from year −28 (0.79, *P* = .001) to year −16 (0.47 kg/m^2^, *P* = .05), starting from year −8 BMI was lower in cases than in control subjects (Table 3).

**Table 3:** Differences in BMI between cases and the others and between cases and control subjects for more than the 28 years before dementia∗

Year	Dementia cases compared with all others		Dementia cases compared with matched control subjects	
	Difference in BMI	*P* value	Difference in BMI	*P* value
−28	0.69	.002	0.79	.001
−27	0.64	.002	0.78	.001
−26	0.60	.004	0.77	.001
−25	0.56	.007	0.76	.001
−24	0.52	.01	0.74	.001
−23	0.47	.02	0.72	.001
−22	0.43	.04	0.70	.002
−21	0.39	.07	0.67	.003
−20	0.35	.11	0.64	.005
−19	0.30	.16	0.60	.009
−18	0.26	.24	0.56	.02
−17	0.22	.33	0.52	.03
−16	0.17	.45	0.47	.05
−15	0.13	.57	0.42	.08
−14	0.09	.71	0.37	.14
−13	0.05	.85	0.31	.22
−12	0.00	.99	0.25	.33
−11	−0.04	.88	0.18	.48
−10	−0.08	.75	0.11	.67
−9	−0.12	.63	0.04	.89
−8	−0.17	.53	−0.04	.88
−7	−0.21	.44	−0.12	.66
−6	−0.25	.36	−0.20	.47
−5	−0.30	.30	−0.29	.31
−4	−0.34	.24	−0.38	.19
−3	−0.38	.20	−0.48	.11
−2	−0.42	.17	−0.58	.06
−1	−0.47	.14	−0.68	.04
0	−0.51	.12	−0.79	.02

Abbreviation: BMI, body mass index.

∗ All analyses adjusted for age, sex, education; analyses also adjusted for their interaction with time and time squared, when *P* < .05.

**Fig. 2:**
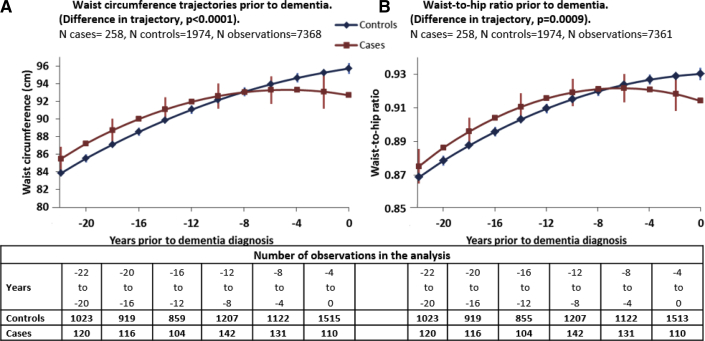
Trajectories of waist circumference (A) and waist-to-hip ratio (B) in the 22 years before dementia. Note: (1) The figure represents marginal effects of dementia on trajectories of waist circumference and waist-to-hip ratio, adjusted for age, sex, and education. Mean number of observations per participant is 2.8 for cases and 3.4 for control subjects. (2) Confidence intervals are presented for alternate time points for ease of presentation. (3) Estimated differences in BMI at each time point are presented in Supplementary Table 4.

Fig. 2 (Panel A) shows waist circumference trajectories for more than the 22 years to differ in cases and control subjects (*P* < .0001); yearly differences in waist circumference are tabulated in Supplementary Table 4. Waist circumference in those with dementia was higher from year −22 (1.64 cm, *P* = .04) to year −16 (1.46, *P* = .04) and lower starting 8 years before dementia. Fig. 2 (Panel B) shows a similar pattern of results for waist-to-hip ratio (Supplementary Table 4 provides yearly differences), with the trajectories in those with dementia being different from the control subjects (*P* = .0009) and rapid decline close to dementia diagnosis.

## 4 Discussion

Results from the Whitehall II study of more than 10,000 men and women with repeat BMI assessments for more than 28 years show that obesity (BMI ≥30 kg/m^2^) at age 50 years is a risk factor for dementia. This association was greatly attenuated when BMI was assessed at ages 60 and 70 years. These findings were corroborated in analysis of trajectories where BMI differences between those with dementia and those free of dementia were examined for each of the 28 years preceding dementia diagnosis. Taken together, the present data demonstrate that the association between obesity and dementia is modified by age at obesity measurement, such that midlife obesity is a risk factor for dementia but BMI begins to decline in those with dementia in the years before diagnosis.

Data from several studies show increased dementia risk with midlife obesity [1], [3], [4], [25], [26], [27], [28], [29], these are reflected in conclusions of meta-analyses [2], [6], [7]. However, a recent large-scale study found obesity to be protective, with the risk of obesity decreasing with increasing levels of obesity such that those in class III obesity (BMI ≥40 kg/m^2^) had 29% (95% confidence interval 22–36) lower risk of dementia than normal weight adults [9]. As the reported analyses were based on only a third of the eligible sample because of missing data on BMI, selection bias is likely to affect results. Furthermore, a follow-up of less than 10 years is perhaps too short a period to remove effects of preclinical dementia, given that changes underlying dementia are known to unfold over many years [15]. In our data, use of a backward timescale on all participants shows the BMI trajectories to intersect 12 years before the diagnosis of dementia, this was reduced to 8 years in analysis using a case-control design, which adjusted for covariates better and took period effects into account.

The risk of dementia associated with obesity at older ages is either attenuated or reversed [30]. This is possibly because of preclinical changes in weight in the years preceding clinical onset of dementia [31], estimated to result in a 10% loss of total body weight [18], and observed in previous studies for more than a period of 2 to 4 years [18], 5 years [32], or up to 10 years [13] before diagnosis of dementia. A combination of predementia apathy, loss of initiative, and reduced olfactory function could explain this association [13]. As evidence of the risk associated with high BMI in midlife and low BMI at older ages comes from separate data sets, the findings on BMI have been interpreted as being conflicting or inconsistent. At least two previous studies used two BMI assessments, one in midlife and the second at older ages [28], [29], and both show higher BMI in midlife and lower BMI in late life to carry risk for dementia. Our study is the first to model BMI trajectories for more than nearly three decades before dementia using an analytic strategy anchored to the etiology of disease. The advantage of this method is that the timescale used in the analysis is years before dementia diagnosis rather than age (which can vary between individuals for dementia onset) or data collection cycle (which conflates the effects of age and stage of dementia).

Two recent MR studies, using overlapping data on older adults [11], [33], showed no association between obesity and dementia. These studies might be affected by selective BMI-related survival [33]. The AD Genetics Consortium, which contributed the bulk of cases and control subjects to both articles, is based on cases where the mean age of dementia onset was 74.7 years and the age of examination of control subjects was 76.3 years. When cases and control subjects are drawn from older adults, the MR approach is subject to “survivor” bias [33], in this case because of higher mortality in the obese [34]. In our data among participants born between 1930 and 1940, 24% of the obese at baseline did not reach age 75 years compared with 15% of their normal weight counterparts.

The exact mechanisms explaining the increased risk of dementia associated with obesity are poorly understood. Obesity in midlife [35] and at older ages [36] is associated with brain atrophy. There is also evidence suggesting a variant of the fat mass and obesity-associated (FTO) gene affects brain structure, causing deficits in the frontal and occipital lobes [37], [38]. Obesity is also likely to influence cognition through its impact on vascular risk factors and pathology, and some authors have highlighted the role of adipocyte hormones and cytokines [16]. Genome-wide association studies, in turn, show genetic risk variants that influence vascular and inflammatory pathways to be associated with dementia [39], [40]. Potential avenues of further research include brain atrophy, disruption in cerebrovascular function, development of amyloid pathology, breakdown in the blood-brain barrier, and systemic and neuroinflammation.

The major strength of our study is the use of BMI data covering a period of 28 years, which allowed us to assess both the risk associated with obesity at specific ages and model trajectories of BMI for more than 28 years before dementia onset. Our use of both BMI and other indices of adiposity at specific ages and their trajectories over the adult life course allow the natural history of this relationship to be established. The case-control design analysis strategy ensured a better control for period effects (the effects of calendar time on dementia diagnosis or adiposity) and confounders (age, sex, and education). The use of three markers of obesity also allows us to conclude that BMI, the most widely used measure because of ease of data collection, is suitable for the assessment of risk of dementia and death.

A key limitation of our study is underascertainment of dementia because of use of linkage to electronic health records. A comparison of the passive case finding via record linkage to an active approach in the Mayo Clinic Study of Aging and the Adult Changes reported the passive approach to have very high specificity and sensitivity of approximately 70%, missing mostly milder cases of dementia [41]. A similar pattern is likely in our study because of universal health coverage in the UK, where electronic health records have been shown to be reliable for dementia [42]. There was no evidence in our data that BMI affected age of dementia diagnosis. In analyses on BMI at 50 years the age of dementia diagnosis was 75.62 years in normal weight participants, 75.64 years in overweight participants, and 75.85 years in obese participants. Thus, any misclassification of dementia status is likely to be random, that is, the probability of dementia status being misclassified is independent of BMI. This means that under conditions of high specificity, the association between risk factor and outcome is unlikely to be biased by underascertainment of the outcome [43]. Furthermore, consistency in results between the survival analysis and that using trajectories of adiposity markers suggests that these results are also robust to nonrandom misclassification. The advantage of our passive case finding approach is that dementia status was available on all participants and not only on those who continued to participate in the study over the follow-up, thus increasing the generalizability of findings.

We were unable to examine the subcategories of dementia because of small numbers but previous reports show similar findings for the association of obesity with dementia subtypes [4], [18], [27], [28], [31]. Finally, the prevalence of obesity was relatively low in this cohort and it was not possible to undertake detailed analyses stratified by degrees of obesity; for example, only 2.2%, 4.1%, and 5.2% of participants were severely obese (BMI >35 kg/m^2^) at ages 50, 60, and 70 years, respectively.

Our findings have important implications. Considerable improvements in cardiovascular health and education over the second half of the last century are suggested to be responsible for the leveling of the incidence rates of dementia [44]. In the past 40 years, however, there has been a startling increase in the number of obese persons, rising from 105 million in 1975 to 641 million in 2014. Our results suggest midlife obesity is a risk factor for dementia, and the extent to which the continuing obesity epidemic will create a surge in future dementia rates is an important public health issue.

**Research in Context**

1 Systematic review. We searched PubMed, to identify the scientific literature on the association between obesity and dementia. Meta-analyses, the last publication dated from 2011 (total *n* = 30, 470), suggest that midlife obesity is associated with increased risk of dementia. However, a 2015 publication on 2 million adults showed lower dementia risk in the obese, with the risk decreasing with increasing obesity. Thus, whether obesity is a risk factor for dementia is unclear despite it being included routinely in guidelines that aim to prevent/delay dementia.
2 Interpretation. Obesity at age 50 years but not at ages 60 and 70 years was associated with increased risk of dementia. Natural history of body mass index (BMI), anchored to dementia diagnosis such that changes in BMI were modeled over 28 years preceding dementia diagnosis, shows obesity in midlife and weight loss in the preclinical phase to characterize BMI changes in those with dementia.
3 Future directions. Obesity in midlife carries risk for dementia; not accounting for the preclinical phase explains inconsistency in results from previous studies. Our results highlight the importance of the analytic framework used to identify putative risk factors for dementia. The ongoing obesity epidemic may well impact dementia incidence in the future.

## References

[1] Whitmer R.A., Gustafson D.R., Barrett-Connor E., Haan M.N., Gunderson E.P., Yaffe K. (2008). Central obesity and increased risk of dementia more than three decades later. Neurology.

[2] Gorospe E.C., Dave J.K. (2007). The risk of dementia with increased body mass index. Age Ageing.

[3] Chuang Y.F., An Y., Bilgel M., Wong D.F., Troncoso J.C., O'Brien R.J. (2016). Midlife adiposity predicts earlier onset of Alzheimer's dementia, neuropathology and presymptomatic cerebral amyloid accumulation. Mol Psychiatry.

[4] Kivipelto M., Ngandu T., Fratiglioni L., Viitanen M., Kareholt I., Winblad B. (2005). Obesity and vascular risk factors at midlife and the risk of dementia and Alzheimer disease. Arch Neurol.

[5] Rosengren A., Skoog I., Gustafson D., Wilhelmsen L. (2005). Body mass index, other cardiovascular risk factors, and hospitalization for dementia. Arch Intern Med.

[6] Beydoun M.A., Beydoun H.A., Wang Y. (2008). Obesity and central obesity as risk factors for incident dementia and its subtypes: a systematic review and meta-analysis. Obes Rev.

[7] Anstey K.J., Cherbuin N., Budge M., Young J. (2011). Body mass index in midlife and late-life as a risk factor for dementia: a meta-analysis of prospective studies. Obes Rev.

[8] Emmerzaal T.L., Kiliaan A.J., Gustafson D.R. (2015). 2003-2013: a decade of body mass index, Alzheimer's disease, and dementia. J Alzheimers Dis.

[9] Qizilbash N., Gregson J., Johnson M.E., Pearce N., Douglas I., Wing K. (2015). BMI and risk of dementia in two million people over two decades: a retrospective cohort study. Lancet Diabetes Endocrinol.

[10] Ostergaard S.D., Mukherjee S., Sharp S.J., Proitsi P., Lotta L.A., Day F. (2015). Associations between potentially modifiable risk factors and Alzheimer disease: a Mendelian randomization study. PLoS Med.

[11] Mukherjee S., Walter S., Kauwe J.S., Saykin A.J., Bennett D.A., Larson E.B. (2015). Genetically predicted body mass index and Alzheimer's disease-related phenotypes in three large samples: Mendelian randomization analyses. Alzheimers Dement.

[12] Winblad B., Amouyel P., Andrieu S., Ballard C., Brayne C., Brodaty H. (2016). Defeating Alzheimer's disease and other dementias: a priority for European science and society. Lancet Neurol.

[13] Knopman D.S., Edland S.D., Cha R.H., Petersen R.C., Rocca W.A. (2007). Incident dementia in women is preceded by weight loss by at least a decade. Neurology.

[14] Nelson P.T., Braak H., Markesbery W.R. (2009). Neuropathology and cognitive impairment in Alzheimer disease: a complex but coherent relationship. J Neuropathol Exp Neurol.

[15] Jack C.R., Knopman D.S., Jagust W.J., Petersen R.C., Weiner M.W., Aisen P.S. (2013). Tracking pathophysiological processes in Alzheimer's disease: an updated hypothetical model of dynamic biomarkers. Lancet Neurol.

[16] Gustafson D. (2006). Adiposity indices and dementia. Lancet Neurol.

[17] Gustafson D.R., Backman K., Joas E., Waern M., Ostling S., Guo X. (2012). 37 years of body mass index and dementia: observations from the prospective population study of women in Gothenburg, Sweden. J Alzheimers Dis.

[18] Stewart R., Masaki K., Xue Q.L., Peila R., Petrovitch H., White L.R. (2005). A 32-year prospective study of change in body weight and incident dementia: the Honolulu-Asia Aging Study. Arch Neurol.

[19] Marmot M.G., Smith G.D., Stansfeld S., Patel C., North F., Head J. (1991). Health inequalities among British civil servants: the Whitehall II Study. Lancet.

[20] WHO (2000). Obesity: Preventing and managing the global epidemic. Report of a WHO consultation. World Health Organ Tech Rep Ser.

[21] Han T.S., van Leer E.M., Seidell J.C., Lean M.E. (1995). Waist circumference action levels in the identification of cardiovascular risk factors: prevalence study in a random sample. BMJ.

[22] (2006). Obesity: the Prevention, Identification, Assessment and Management of Overweight and Obesity in Adults and Children. London.

[23] Singh-Manoux A., Kivimaki M., Glymour M.M., Elbaz A., Berr C., Ebmeier K.P. (2012). Timing of onset of cognitive decline: results from Whitehall II prospective cohort study. BMJ.

[24] Amieva H., Le G.M., Millet X., Orgogozo J.M., Peres K., Barberger-Gateau P. (2008). Prodromal Alzheimer's disease: successive emergence of the clinical symptoms. Ann Neurol.

[25] Xu W.L., Atti A.R., Gatz M., Pedersen N.L., Johansson B., Fratiglioni L. (2011). Midlife overweight and obesity increase late-life dementia risk: a population-based twin study. Neurology.

[26] Whitmer R.A., Gunderson E.P., Barrett-Connor E., Quesenberry C.P., Yaffe K. (2005). Obesity in middle age and future risk of dementia: a 27 year longitudinal population based study. BMJ.

[27] Whitmer R.A., Gunderson E.P., Quesenberry C.P., Zhou J., Yaffe K. (2007). Body mass index in midlife and risk of Alzheimer disease and vascular dementia. Curr Alzheimer Res.

[28] Tolppanen A.M., Ngandu T., Kareholt I., Laatikainen T., Rusanen M., Soininen H. (2014). Midlife and late-life body mass index and late-life dementia: results from a prospective population-based cohort. J Alzheimers Dis.

[29] Fitzpatrick A.L., Kuller L.H., Lopez O.L., Diehr P., O'Meara E.S., Longstreth W.T. (2009). Midlife and late-life obesity and the risk of dementia: cardiovascular health study. Arch Neurol.

[30] Luchsinger J.A., Patel B., Tang M.X., Schupf N., Mayeux R. (2007). Measures of adiposity and dementia risk in elderly persons. Arch Neurol.

[31] Hughes T.F., Borenstein A.R., Schofield E., Wu Y., Larson E.B. (2009). Association between late-life body mass index and dementia: the Kame Project. Neurology.

[32] Buchman A.S., Wilson R.S., Bienias J.L., Shah R.C., Evans D.A., Bennett D.A. (2005). Change in body mass index and risk of incident Alzheimer disease. Neurology.

[33] Boef A.G., le Cessie S., Dekkers O.M. (2015). Mendelian randomization studies in the elderly. Epidemiology.

[34] Whitlock G., Lewington S., Sherliker P., Clarke R., Emberson J., Halsey J. (2009). Body-mass index and cause-specific mortality in 900 000 adults: collaborative analyses of 57 prospective studies. Lancet.

[35] Debette S., Beiser A., Hoffmann U., DeCarli C., O'Donnell C.J., Massaro J.M. (2010). Visceral fat is associated with lower brain volume in healthy middle-aged adults. Ann Neurol.

[36] Ho A.J., Raji C.A., Becker J.T., Lopez O.L., Kuller L.H., Hua X. (2010). Obesity is linked with lower brain volume in 700 AD and MCI patients. Neurobiol Aging.

[37] Ho A.J., Stein J.L., Hua X., Lee S., Hibar D.P., Leow A.D. (2010). A commonly carried allele of the obesity-related FTO gene is associated with reduced brain volume in the healthy elderly. Proc Natl Acad Sci U S A.

[38] Debette S., Wolf C., Lambert J.C., Crivello F., Soumare A., Zhu Y.C. (2014). Abdominal obesity and lower gray matter volume: a Mendelian randomization study. Neurobiol Aging.

[39] Tosto G., Reitz C. (2013). Genome-wide association studies in Alzheimer's disease: a review. Curr Neurol Neurosci Rep.

[40] Lambert J.C., Ibrahim-Verbaas C.A., Harold D., Naj A.C., Sims R., Bellenguez C. (2013). Meta-analysis of 74,046 individuals identifies 11 new susceptibility loci for Alzheimer's disease. Nat Genet.

[41] Knopman D.S., Petersen R.C., Rocca W.A., Larson E.B., Ganguli M. (2011). Passive case-finding for Alzheimer's disease and dementia in two U.S. communities. Alzheimers Dement.

[42] Brown A., Kirichek O., Balkwill A., Reeves G., Beral V., Sudlow C. (2016). Comparison of dementia recorded in routinely collected hospital admission data in England with dementia recorded in primary care. Emerg Themes Epidemiol.

[43] Copeland K.T., Checkoway H., McMichael A.J., Holbrook R.H. (1977). Bias due to misclassification in the estimation of relative risk. Am J Epidemiol.

[44] Larson E.B., Yaffe K., Langa K.M. (2013). New insights into the dementia epidemic. N Engl J Med.

