# Supplementary material for: Obesity trajectories and risk of dementia: 28 years of follow-up in the Whitehall II Study
Source: Alzheimers Dement. 2018 Feb;14(2):178–86. doi: 10.1016/j.jalz.2017.06.2637 (PMC5805839; doi:10.1016/j.jalz.2017.06.2637)
Supplement: Supplementary Tables 1–4 and Figures 1 and 2 [file mmc1.docx]

**SUPPLEMENTAL MATERIALS**

**Obesity trajectories over 28 years and risk of dementia: a longitudinal study**

Archana Singh-Manoux, Aline Dugravot, Martin Shipley, Eric J. Brunner, Alexis Elbaz, Séverine Sabia, Mika Kivimaki

Table S1. Association of BMI at ages 50, 60 and 70 years with subsequent dementia and mortality.

Table S2. Association of waist circumference and waist-to-hip ratio at ages 50, 60 and 70 years with subsequent dementia and mortality, minimally adjusted models.

Table S3. Association of waist circumference and waist-to-hip ratio at ages 50, 60 and 70 years with subsequent dementia and mortality, fully adjusted models.

Table S4. Differences in Waist Circumference (WC) and Waist-to-Hip Ratio (WHR) between cases and controls over the 22 years prior dementia.

Figure S1. Study flow chart of sample selection, Whitehall II Study.

Figure S2. Trajectory of global cognitive score in dementia cases and other participants in the years leading to dementia diagnosis.

**Table S1. Association of BMI at ages 50, 60 and 70 years with subsequent dementia and mortality.^†^**

|  |  | **Dementia** | | |  | **Mortality** | | |
| --- | --- | --- | --- | --- | --- | --- | --- | --- |
|  |  | **N cases/N** | **HR (95%CI)** | **P** |  | **N cases/N** | **HR (95%CI)** | **P** |
| **BMI at 50 years** |  |  |  |  |  |  |  |  |
| < 18.5 Kg/m^2^ |  | 1/72 | 0.52 (0.07-3.69) |  |  | 13/72 | 1.44 (0.83-2.50) | 0.19 |
| 18.5-24.9 Kg/m^2^ |  | 128/4208 | 1.00 | Ref |  | 611/4208 | 1.00 | Ref |
| 25-29.9 Kg/m^2^ |  | 104/3219 | 1.17 (0.90-1.52) | 0.23 |  | 505/3219 | 1.10 (0.98-1.24) | 0.10 |
| ≥30 Kg/m^2^ |  | 42/913 | 1.88 (1.32-2.69) | 0.001 |  | 176/913 | 1.54 (1.30-1.83) | <0.0001 |
| **BMI at 60 years** |  |  |  |  |  |  |  |  |
| < 18.5 Kg/m^2^ |  | 0/63 | NA |  |  | 12/63 | 2.52(1.41- 4.50) | 0.002 |
| 18.5-24.9 Kg/m^2^ |  | 98/2819 | 1 | Ref |  | 343/2819 | 1.00 | Ref |
| 25-29.9 Kg/m^2^ |  | 96/3162 | 0.96 (0.72-1.28) | 0.79 |  | 388/3162 | 1.07 (0.92-1.23) | 0.40 |
| ≥30 Kg/m^2^ |  | 38/1246 | 1.15 (0.77-1.70) | 0.50 |  | 169/1246 | 1.32 (1.09-1.60) | 0.005 |
| **BMI at 70 years** |  |  |  |  |  |  |  |  |
| < 18.5 Kg/m^2^ |  | 2/39 | 1.62 (0.39-6.78) |  |  | 6/39 | 2.16 (0.94-4.95) | 0.07 |
| 18.5-24.9 Kg/m^2^ |  | 72/1731 | 1.00 | Ref |  | 166/1734 | 1.00 | Ref |
| 25-29.9 Kg/m^2^ |  | 57/2159 | 0.59 (0.42-0.85) | 0.004 |  | 196/2160 | 0.89 (0.72-1.10) | 0.28 |
| ≥30 Kg/m^2^ |  | 30/904 | 0.80 (0.51-1.25) | 0.33 |  | 102/907 | 1.23 (0.95-1.60) | 0.11 |

^†^Analyses adjusted for age, sex, education, diabetes, CVD and CVD medication.

**Table S2. Association of waist circumference and waist-to-hip ratio at ages 50, 60 and 70 years with subsequent dementia and mortality, minimally adjusted models.^†^**

|  |  | **Dementia** | | |  | **Mortality** | | | |
| --- | --- | --- | --- | --- | --- | --- | --- | --- | --- |
|  |  | **N cases/N** | **HR (95%CI)** | **p** |  | **N cases/N** | **HR (95%CI)** | **P** | |
| **Waist Circumference (cm) at 50 years (Men/Women)** | | | | | | | | |  |
| ≤ 94/≤80 women18.5 Kg/m^2^ |  | 41/3354 | 1.00 | Ref |  | 272/3354 | 1.00 | Ref | |
| 94-102/80-88 Kg/m^2^ |  | 15/1038 | 1.32 (0.73-2.39) | 0.35 |  | 90/1038 | 1.12 (0.88-1.42) | 0.35 | |
| >102/>88 |  | 14/830 | 1.80 (0.98-3.33) | 0.06 |  | 104/830 | 1.82 (1.44-2.29) | <0.0001 | |
| **Waist Circumference (cm) at 60 years (Men/Women)** | | | | | | | | |  |
| ≤ 94/≤80 women18.5 Kg/m^2^ |  | 115/3303 | 1.00 | Ref |  | 417/3303 | 1.00 | Ref | |
| 94-102/80-88 Kg/m^2^ |  | 49/1804 | 1.02 (0.73-1.44) | 0.89 |  | 201/1804 | 1.11 (0.94-1.32) | 0.23 | |
| >102/>88 |  | 50/1987 | 1.30 (0.92-1.84) | 0.14 |  | 227/1987 | 1.49 (1.26-1.76) | <0.0001 | |
| **Waist Circumference (cm) at 70 years (Men/Women)** | | | | | | | | |  |
| ≤ 94/≤80 women18.5 Kg/m^2^ |  | 77/1720 | 1.00 | Ref |  | 164/1723 | 1.00 | Ref | |
| 94-102/80-88 Kg/m^2^ |  | 42/1325 | 0.72 (0.49-1.05) | 0.09 |  | 112/1328 | 0.95 (0.74-1.20) | 0.65 | |
| >102/>88 |  | 42/1846 | 0.57 (0.39-0.85) | 0.005 |  | 173/1848 | 1.27 (1.02-1.59) | 0.03 | |
| **Waist-to-hip ratio at 50 years (Men/Women)** | | | | | | | | |  |
| <1/<0.85 |  | 58/4363 | 1.00 | Ref |  | 353/4363 | 1.00 | Ref | |
| ≥1/≥0.85 |  | 12/849 | 1.16 (0.62-2.19) | 0.65 |  | 113/849 | 1.82 (1.46-2.26) | <0.0001 | |
| **Waist-to-hip ratio at 60 years (Men/Women)** | | | | | | | | |  |
| <1/<0.85 |  | 156/5045 | 1.00 | Ref |  | 569/5045 | 1.00 | Ref | |
| ≥1/≥0.85 |  | 58/2043 | 1.21 (0.88-1.66) | 0.24 |  | 276/2043 | 1.59 (1.37-1.85) | <0.0001 | |
| **Waist-to-hip ratio at 70 years (Men/Women)** | | | | | | | | |  |
| <1/<0.85 |  | 106/3022 | 1.00 | Ref |  | 274/3026 | 1.00 | Ref | |
| ≥1/≥0.85 |  | 55/1865 | 0.84 (0.60-1.18) | 0.32 |  | 174/1869 | 1.14 (0.94-1.39) | 0.19 | |

^†^Analyses adjusted for age, sex, and education.

**Table S3. Association of waist circumference and waist-to-hip ratio at ages 50, 60 and 70 years with subsequent dementia and mortality, fully adjusted models.^†^**

|  |  | **Dementia** | | |  | **Mortality** | | | |
| --- | --- | --- | --- | --- | --- | --- | --- | --- | --- |
|  |  | **N cases/N** | **HR (95%CI)** | **P** |  | **N cases/N** | **HR (95%CI)** | **P** | |
| **Waist Circumference (cm) at 50 years (Men/Women)** | | | | | | | | |  |
| ≤ 94/≤80 women18.5 Kg/m^2^ |  | 41/3354 | 1.00 | Ref |  | 272/3354 | 1.00 | Ref | |
| 94-102/80-88 Kg/m^2^ |  | 15/1038 | 1.28 (0.71-2.32) | 0.42 |  | 90/1038 | 1.09 (0.86-1.38) | 0.49 | |
| >102/>88 |  | 14/830 | 1.72 (0.92-3.21) | 0.08 |  | 104/830 | 1.68 (1.33-2.13) | <0.0001 | |
| **Waist Circumference (cm) at 60 years (Men/Women)** | | | | | | | | |  |
| ≤ 94/≤80 women18.5 Kg/m^2^ |  | 115/3303 | 1.00 | Ref |  | 417/3303 | 1.00 | Ref | |
| 94-102/80-88 Kg/m^2^ |  | 49/1804 | 0.95 (0.68-1.34) | 0.79 |  | 201/1804 | 1.04 (0.88-1.23) | 0.65 | |
| >102/>88 |  | 50/1987 | 1.15 (0.80-1.64) | 0.44 |  | 227/1987 | 1.32 (1.11-1.57) | 0.002 | |
| **Waist Circumference (cm) at 70 years (Men/Women)** | | | | | | | | |  |
| ≤ 94/≤80 women18.5 Kg/m^2^ |  | 77/1720 | 1.00 | Ref |  | 164/1723 | 1.00 | Ref | |
| 94-102/80-88 Kg/m^2^ |  | 42/1325 | 0.68 (0.46-0.99) | 0.05 |  | 112/1328 | 0.87 (0.69-1.12) | 0.28 | |
| >102/>88 |  | 42/1846 | 0.52 (0.35-0.78) | 0.002 |  | 173/1848 | 1.14 (0.90-1.43) | 0.27 | |
| **Waist-to-hip ratio at 50 years (Men/Women)** | | | | | | | | |  |
| <1/<0.85 |  | 58/4363 | 1.00 | Ref |  | 353/4363 | 1.00 | Ref | |
| ≥1/≥0.85 |  | 12/849 | 1.12 (0.59-2.11) | 0.74 |  | 113/849 | 1.68 (1.35-2.10) | <0.0001 | |
| **Waist-to-hip ratio at 60 years (Men/Women)** | | | | | | | | |  |
| <1/<0.85 |  | 156/5045 | 1.00 | Ref |  | 569/5045 | 1.00 | Ref | |
| ≥1/≥0.85 |  | 58/2043 | 1.08 (0.79-1.49) | 0.63 |  | 276/2043 | 1.44 (1.24-1.68) | <0.0001 | |
| **Waist-to-hip ratio at 70 years (Men/Women)** | | | | | | | | |  |
| <1/<0.85 |  | 106/3022 | 1.00 | Ref |  | 274/3026 | 1.00 | Ref | |
| ≥1/≥0.85 |  | 55/1865 | 0.79 (0.55-1.11) | 0.18 |  | 174/1869 | 1.02 (0.83-1.25) | 0.83 | |

^†^Analyses adjusted for age, sex, education, diabetes, CVD and CVD medication.

**Table S4. Differences in Waist Circumference (WC) and Waist-to-Hip Ratio (WHR) between cases and controls over the 22 years prior dementia.**^†^

For accompanying trajectories see Figure 2.

| **Year** | **Difference in WC** | **p** |  | **Difference in WHR** | **P** |
| --- | --- | --- | --- | --- | --- |
| -22 | 1.64 | 0.04 |  | 0.007 | 0.24 |
| -21 | 1.67 | 0.03 |  | 0.007 | 0.15 |
| -20 | 1.68 | 0.02 |  | 0.008 | 0.10 |
| -19 | 1.66 | 0.02 |  | 0.008 | 0.07 |
| -18 | 1.61 | 0.02 |  | 0.008 | 0.06 |
| -17 | 1.55 | 0.03 |  | 0.008 | 0.05 |
| -16 | 1.46 | 0.04 |  | 0.008 | 0.06 |
| -15 | 1.35 | 0.06 |  | 0.008 | 0.07 |
| -14 | 1.22 | 0.09 |  | 0.007 | 0.09 |
| -13 | 1.06 | 0.15 |  | 0.007 | 0.12 |
| -12 | 0.88 | 0.23 |  | 0.006 | 0.17 |
| -11 | 0.68 | 0.36 |  | 0.005 | 0.25 |
| -10 | 0.46 | 0.54 |  | 0.004 | 0.37 |
| -9 | 0.21 | 0.78 |  | 0.003 | 0.54 |
| -8 | -0.06 | 0.94 |  | 0.001 | 0.77 |
| -7 | -0.35 | 0.65 |  | 0.000 | 0.95 |
| -6 | -0.67 | 0.40 |  | -0.002 | 0.65 |
| -5 | -1.01 | 0.21 |  | -0.004 | 0.39 |
| -4 | -1.37 | 0.10 |  | -0.006 | 0.21 |
| -3 | -1.75 | 0.04 |  | -0.008 | 0.10 |
| -2 | -2.16 | 0.02 |  | -0.011 | 0.05 |
| -1 | -2.59 | 0.007 |  | -0.013 | 0.03 |
| 0 | -3.04 | 0.003 |  | -0.016 | 0.02 |

^†^All analyses adjusted for age, sex, education, analyses also adjusted for their interaction with time and time² when p<0.05.

**Figure S1. Study flow chart of sample selection, Whitehall II Study.**

**1985-1988**

Age 35-55 years

Adiposity measures, N=10293

**1991-1993**

Age 40-64 years

Adiposity measures, N =8074

**1997-1999**

Age 45-69 years

Adiposity measures, N =5681

**2002-2004**

Age 50-74 years

Adiposity measures, N =6450

**2007-2009**

Age 55-79 years

Adiposity measures, N =6193

**2012-2013**

Age 60-83 years

Adiposity measures, N =5615

**Incident cases**

N dementia=0

N mortality=94

**Incident cases**

N dementia=1

N mortality=181

**Incident cases**

N dementia=14

N mortality=270

**Incident cases**

N dementia=43

N mortality=348

**Incident cases**

N dementia=137

N mortality=435

**End of follow-up: 2015**

**Total number of cases:**

**N dementia=329**

**N mortality=1653**

**Incident cases**

N dementia=134

N mortality=325

**Figure S2. Trajectory of global cognitive score in dementia cases and other participants in the years leading to dementia diagnosis.**

**Difference in trajectory, p<0.0001**

|  | **Number of observations in the analysis** | | | | | |
| --- | --- | --- | --- | --- | --- | --- |
| **Years** | | **-18 to -16** | **-16 to -12** | **-12 to-8** | **-8 to-4** | **-4 to 0** |
| **Dementia free (N=7311)** | | 5192 | 2489 | 3714 | 5938 | 4777 |
| **Dementia cases (N=195)** | | 31 | 81 | 119 | 110 | 89 |
